# Development and reliability testing of a risk factor and risk outcome assessment scale for nurses in “internet + nursing services” for the elderly

**DOI:** 10.1186/s12912-024-01698-2

**Published:** 2024-01-19

**Authors:** Jiajia Xu, Yuping Shi, Shan Li, Jinglian Ma, Jianghong Zhang, Yanfang Shen

**Affiliations:** 1 General Medical Department, Third Hospital of Shanxi Medical University, Shanxi Bethune Hospital, Shanxi Academy of Medical Sciences, Tongji Shanxi Hospital, Taiyuan City, Shanxi Province 030032 China; 2 College of Nursing, Shanxi Medical University, Taiyuan City, Shanxi Province 030001 China

**Keywords:** Nurses, Risk perception, Risk factors, Scales, “Internet + nursing services”

## Abstract

**Background:**

China is experiencing an aging population, leading to a significant demand for “Internet + nursing services” tailored for elderly individuals. However, there are many risk problems in the process of nurse service, which hinder the development of the service, and a scale is needed to assess the risk problems faced by nurses in “Internet + nursing services” for the elderly.

**Objective:**

The purpose of this study is to develop an assessment scale for risk factors and outcomes related to nurses’ involvement in the “Internet + Nursing Service” for the elderly and to assess its reliability and validity.

**Methods:**

Based on literature analysis, focus group, the Delphi method, and a presurvey, we designed an initial scale. The initial scale comprised two sections: risk factors and risk outcomes for nurses. In January and February of 2023, nurses engaged in “Internet + nursing services” for the elderly in Shanxi Province were chosen through a convenience sampling technique for a questionnaire survey. Subsequently, item analysis and exploratory factor analysis were employed to refine and develop a test version of the scale further. A follow-up questionnaire survey was carried out in March and April 2023 using a similar approach. The reliability and validity of the scale were assessed through confirmatory factor analysis, culminating in the formation of the final scale.

**Results:**

The initial survey yielded 244 valid responses. The cumulative variance contributions of the two segments from the exploratory factor analysis were 84.584% and 90.089%, respectively. A subsequent survey garnered 220 valid responses. The confirmatory factor analysis results indicated: χ2/df = 2.086, comparative fit index (CFI) = 0.918, normative fit index (NLI) = 0.855, root mean square of residuals (RMR) = 0.045, and root mean square of error of approximation (RMSEA) = 0.070. These results demonstrate good structural, convergent, and discriminant validity. The content validity index at the item level (I-CVI) ranged between 0.875 and 1.000, while the content validity index at the scale level (S-CVI/Ave) was 0.941. Cronbach’s alpha coefficient for the entire scale stood at 0.970. Moreover, the scale exhibited a split-half reliability of 0.876 and a retest reliability of 0.980 (*p* < 0.01).

**Conclusion:**

The risk factors and risk outcomes associated with nurses involved in “Internet + nursing services” for elderly individuals, as developed in this study, demonstrate strong reliability and validity. They are well suited to the Chinese national context.

## Introduction

The term “Internet + nursing service” predominantly denotes the care services offered by medical facilities for discharged patients or particular groups with health issues and mobility challenges, utilizing the Internet and adopting an “online application and offline service” approach. Currently, this model has shown pronounced benefits in enhancing people’s lives. Over 2,000 medical institutions nationwide have delivered more than 60 door-to-door medical services for elderly individuals with mobility issues. However, by 2023, the Chinese population aged over 60 has grown to 260 million. Studies indicate that upward of 85% of this age group require varying levels of home care. However, given the emerging nature of this service sector and the associated transitional environment, there remain gaps in service pricing, governance mechanisms, legislative frameworks, and health insurance systems [1–3], exposing nurses to multiple risks.

Nursing risk refers to the legal and economic liability faced by nurses due to medical mistakes during nursing activities [4]. However, in the context of “Internet + nursing service,” we must also consider the personal safety risk faced by nurses, which aligns with policy concerns. The “Internet + Nursing Service” Pilot Work Plan, issued by the Shanghai Municipal Government, emphasizes that the safety of nurses and their medical activities is crucial during the pilot process [5].

Previous research by scholars both at home and abroad has mainly focused on theoretical aspects of risk, such as exploring risk countermeasures, creating evaluation index systems, and developing questionnaires for cross-sectional studies [6–8]. Practical efforts have included providing nurses with risk response training and alarm equipment [9, 10]. However, there is a lack of specific assessment tools for nurses’ service risks, and the risk management system is not yet comprehensive. This situation has led to nurses having concerns about the “Internet + nursing service,” affecting their willingness to participate [11]. Therefore, it is vital to develop scientific risk assessment tools that accurately identify risk factors, assess the level of risk, and provide a foundation for effective risk warnings and countermeasure development. These measures can help improve the risk management system and facilitate the advancement of this service.

The purpose of this study is twofold: (1) to develop an assessment scale for risk factors and outcomes pertaining to nurses engaged in “Internet + Nursing Service” for the elderly and (2) to evaluate the scale’s reliability and validity. and provide an important reference for investigating risk mitigation strategies and to enhance the risk management system of “Internet + Nursing Service.”

## Materials and methods

**Fig. 1:**
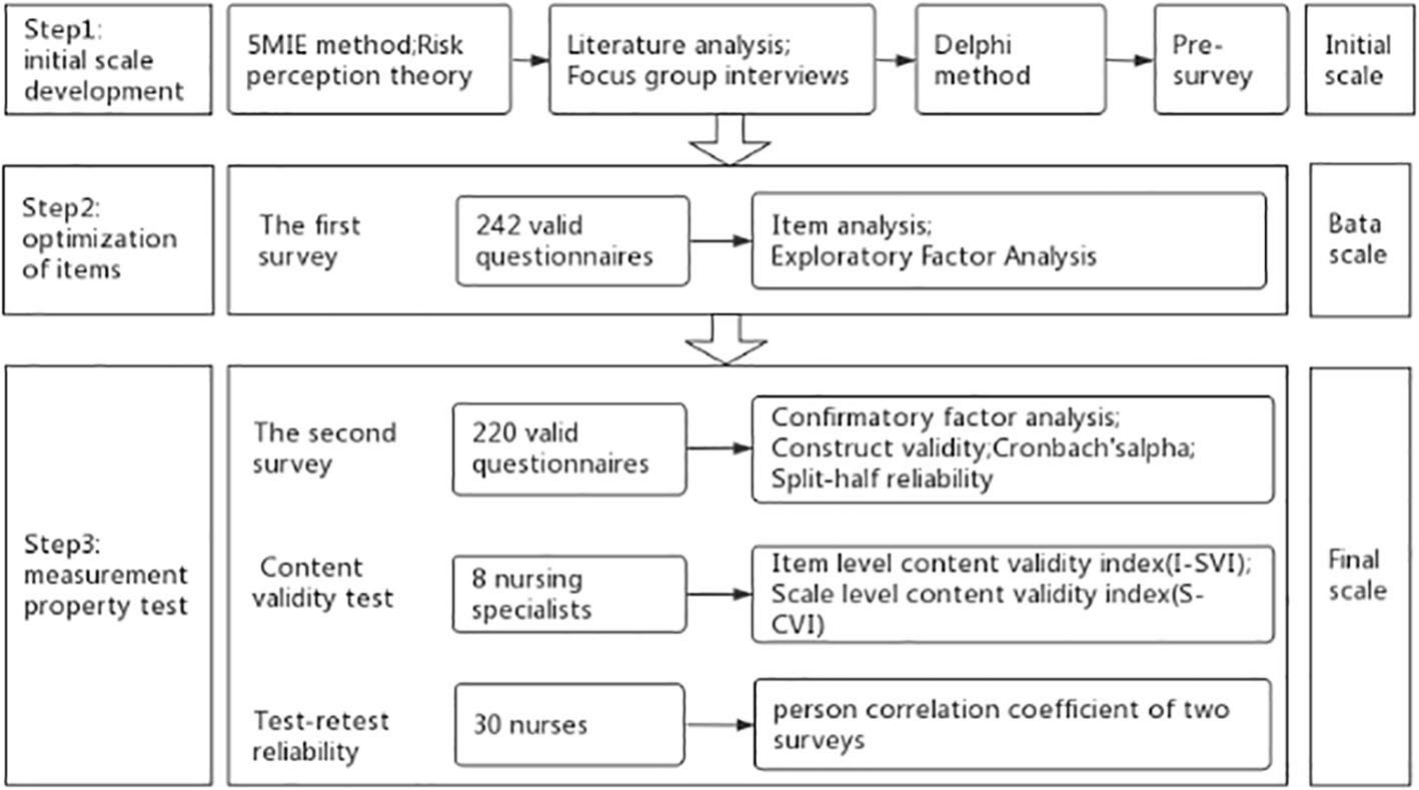
Scale development process

Following the recommendations outlined in the DeVellis scale development procedure [12] and in accordance with the COSMIN guidelines [13], the scale development process is divided into three stages: initial scale formulation, optimization of scale entries, and validation of scale measurement attributes. These phases are sequentially validated as depicted in Fig. 1.

### Initial scale formation

#### Research team formation

A research team was formed, comprising one nursing manager, two deputy chief nursing officers, five supervisory nursing officers, and two nursing graduate students. All team members had experience in geriatric nursing and had engaged in “Internet + nursing services”.

#### Scale entry pool formation

##### Rationale

This research examines the risks that nurses encounter during the service process, which can be categorized as actual risks (those that have occurred and can be objectively described) and potential risks (those that have not yet happened, also known as potential actual risks) [14, 15]. These risks create significant concerns for nurses and can impact their involvement in “Internet+nursing services”. To better understand these risks, the study adopts risk perception theory as the underlying framework. This theory allows us to explore both the objective and potential risks faced by nurses during the service process, providing a visual representation of risk occurrences and perceptions. It serves as an evaluation tool to objectively manage the risks in the service outcomes. In recent years, risk perception theory has gained attention in the medical field. It involves how patients, hospitals, and the entire health system evaluate the extent of risk and make assessments and decisions regarding that risk [16, 17].

This study also examined the actual factors influencing the risks faced by nurses. To do this, we employed the 5M1E method, which includes considering aspects such as man, machine, material, method, measurement, and environment site management theory [18]. By applying this approach to nurse home care as a field service, we were able to comprehensively explore the various dimensions of risk factors nurses encounter during their service process.

##### Literature analysis

In PubMed, Embase, Web of Science, and other English databases, as well as CHKI, VIP, and other Chinese databases, we conducted literature searches using keywords such as “aged,” “elderly,” “old adults,” “geriatric,” “Internet plus nursing service,” “home care,” “home visiting nurse,” “nursing risk,” “risk factor,” “risk assessment,” “5M1E method,” “scale,” and “risk perception theory.” We selected literature relevant to the topic, read the full texts, and extracted information on risk types and factors associated with “Internet + nursing service.”

##### Focus group discussions and establishment of the entry pool

In the context of nursing, risk perception theory encompasses various dimensions, including patients’ economic risk, physical treatment risk, and psychosocial risk [19]. Nurses, due to their different perspectives, face different levels of practice risk [6]. Through focus group discussions, the partial dimensions of nurses’ risk perception include physical risk, psychological risk, practice risk, and economic risk.

Taking into account the specific context of the service, we identified several dimensions of nurse risk factors, including risk assessment related to the patient/family side, risk assessment related to the external environment, risk assessment related to the platform/hospital supply, nurses’ experience in providing service, and the evaluation of nurses’ clinical core competencies.

To present the findings in a reader-friendly manner, the nurse risk factors are organized as the first part of the scale, while the second part of the scale is referred to as the “nurse risk outcome,” which reflects the nurses’ perception of the risks they face during their service. This pool encompassed 9 dimensions and 52 items

#### Delphi method

During October-November 2022, two sessions of expert consultations were carried out. The number of experts was chosen through e-mail, aligning with Guoxiang Xu’s [20] suggested range of 8–20 participants. This study utilized a purposive sampling technique, ultimately selecting 15 experts for consultation. Criteria for expert selection included a minimum of 10 years in geriatric nursing, geriatric nursing education, or management; expertise in managing “Internet + nursing services” for elderly individuals; holding at least a bachelor’s degree; holding a position as a supervising nurse practitioner or higher; and a willingness to participate and actively engage in two or more consultation rounds. Following the initial round of consultation, the research team integrated expert feedback, item selection criteria, and group deliberations. The subsequent consultation questionnaire was then compiled from these results, distributed two weeks later, and gathered within a week.

Post-collection, the experts’ demographic details were tallied, and metrics such as the positivity coefficient, authority coefficient, and opinion alignment degree were computed. The positivity coefficient, reflecting the proportion of effectively returned questionnaires, is given by the formula: effective questionnaires returned/total questionnaires dispatched × 100%. The authority coefficient, Cr, is derived from the expert’s evaluative judgment of scale items (Ca) and their self-rated familiarity (Cs), such that Cr = (Ca + Cs)/2. Kendall’s W and the coefficient of variation (CV) were indicators of the consistency of expert opinions. A higher Kendall’s W indicates more unanimous opinions among the experts. Selection criteria for the indexes were set at an expert opinion and importance score > 4, a coefficient of variation < 0.25, and a full score rate > 40% [21].

#### Pre-survey

Following two expert consultation sessions aimed at refining the item pool, a preliminary survey was initiated. In December 2022, a convenience sampling approach was employed to survey 20 nurses from a tertiary care hospital in Shanxi Province who had experience with “Internet + nursing services” for elderly individuals. This was done to grasp the semantic clarity of the scale and to ascertain if any items were ambiguous. The participants were selected based on the following criteria: (1) nurses who had dedicated ≥ 6 months to professional geriatric nursing; (2) nurses who were certified for “Internet + nursing service” as per their institution’s regulations; (3) nurses who had hands-on experience with “Internet + nursing service” for elderly patients; and (4) nurses who provided informed consent and expressed their willingness to participate in the study.

### Optimization of scale entries and formation of a test version of the scale

This is a quantitative-cross sectional study. Between January and February 2023, the research team undertook a preliminary survey of nursing personnel across level 3, level 2, and level 1 hospitals, as well as those associated with the online platform for “Internet + care service” in Shanxi Province, employing a convenience sampling technique. Both the Questionnaire Star digital platform and paper-based questionnaires were utilized for data collection. Researchers who had undergone questionnaire training were responsible for distributing these tools. The survey instruments encompassed a basic information form for the nursing staff and an initial scale designed to evaluate risk factors and outcomes associated with the “Internet + nursing service” for older adults.

#### Item analysis

The scale entries were analyzed and refined to create the final test version of the scale using three methods: the critical ratio method, correlation coefficient method, and Cronbach’s alpha coefficient method. To evaluate item discrimination, the critical ratio method was applied, and subjects were divided into high and low groups based on their total scores, with the top 27% and bottom 27%, respectively. Entries with a p-value greater than 0.05 were considered for removal [22]. Pearson’s correlation coefficient method was used to examine the relationship between each entry and the total score of the scale. Entries with correlation coefficients of ≥ 0.4 with the total score were retained [23]. Cronbach’s α coefficient method is used to assess the internal consistency of a scale. If removing an item from the total scale results in an increase in Cronbach’s α coefficient, it suggests that the item may impact the scale’s internal consistency, and thus, consideration should be given to its deletion.

#### Exploratory factor analysis

Once the initial entries were evaluated, exploratory factor analysis was employed to assess the structure of the scale. The suitability of the data for factor analysis was determined using the KMO test and Bartlett’s sphericity test, with KMO > 0.8 generally considered appropriate for exploratory factor analysis [22]. We employed principal component analysis for factor extraction and varimax orthogonal rotation. The determination of the number of factors was based on the method of limiting the extraction of common factors, which takes into account the research theory, the study’s purpose, and the examination of the scree plot, and eigenvalues. Entries were eliminated if they met any of the following criteria: (1) entry loadings < 0.5; (2) entries showing similar loading patterns on two or more factors; and (3) fewer than three entries loading under a specific factor. Based on the results of item analysis and exploratory factor analysis, entries that did not meet the scale’s measurement properties were removed to create the version of the test scale.

### Conducting a test of measurement properties and forming the final version of the scale

#### Research subjects

During March-April 2023, the research team conducted a survey using the convenience sampling method. The survey included a basic information questionnaire for nurses and the test version of the scale. The participants were nursing staff from Level 3, Level 2, and Level 1 healthcare facilities in Shanxi Province, as well as those engaged in the “Internet + Nursing Service” through the online platform.

#### Reliability assessment

To assess reliability, three methods were employed: Cronbach’s α coefficient, split-half reliability, and retest reliability. Cronbach’s α coefficient was calculated for both the entire scale and each dimension to determine internal consistency, with a general requirement of Cronbach’s α ≥ 0.7 [24]. For split-half reliability, the correlation coefficient was computed based on scores obtained by subjects on two halves of the questions, and a split-half reliability of ≥ 0.7 was considered acceptable. Retest reliability was evaluated by selecting 30 nurses from the internal medicine of a tertiary hospital. They were asked to complete the same questionnaire again after 2–3 weeks. The data obtained from the two administrations were assessed using Pearson’s correlation coefficient. A correlation coefficient > 0.75 indicated good retest reliability, a correlation coefficient of 0.4–0.75 indicated acceptable retest reliability, and a correlation coefficient < 0.4 indicated poor retest reliability [19].

#### Validity assessment

##### Content validity

For the validity assessment in this study, eight geriatric professional nursing experts were selected. The experts were chosen based on the same criteria as the 2 rounds of expert correspondence. The item content validity index (I-CVI) and scale content validity index (S-CVI) were calculated using the expert scores. The I-CVI was determined by dividing the number of experts who gave a score of 3 or 4 by the total number of participating experts. Similarly, the S-CVI/Ave was calculated as the mean of I-CVI for all entries of the scale. Generally, I-CVI ≥ 0.78 and S-CVI/Ave ≥ 0.9 were considered acceptable [23].

##### Assessment of structural validity validation, convergent validity, and discriminant validity using confirmatory factor analysis

Confirmatory factor analysis was employed to assess the structure of the scale. A good model fit is indicated by a root squared error of approximation (RMSEA) of < 0.05, while a value of 0.05 to 0.08 suggests a basically acceptable fit. The χ2/df value should be < 3. For model fit indices, a value-added fit index (IFI), comparative fit index (CFI), and non-normative fit index (TLI) > 0.9 indicate a good fit, with higher values closer to 1 indicating a better fit. A goodness-of-fit index (GFI) > 0.9 indicates an acceptable fit [25]. Additionally, the correlation coefficient between the scale entries and their respective dimensions should fall between 0.3 and 0.8 [26]. Aggregate validity was analyzed using two indicators: average variance extracted (AVE) and combined reliability (CR). AVE values greater than 0.5 and CR values greater than 0.7 for each factor indicate good aggregate validity [27]. To assess discriminant validity, the square root of AVE values was compared with the correlation coefficients of other factors. If the square root of AVE values is greater than the correlation coefficients between the factor and other factors, it indicates good discriminant validity [28].

### Data collection and quality control

The initial and resurvey were conducted using a self-developed general information questionnaire along with the initial scale and the test version of the scale, respectively. Both electronic and paper versions of the questionnaire were utilized. Electronic questionnaires were distributed via the Internet, accessible through cell phones, computers, and other electronic devices. Before answering the questions, the research subjects needed to agree to an informed consent form presented on the first page of the questionnaire. They had the freedom to quit answering at any time. All questions were marked as needed fields to ensure the completeness of the questionnaire. Additionally, measures were taken to prevent repeated filling by the same individual using the same micro signal. To ensure data quality, electronic questionnaires were manually checked. Entries with a response time of less than 120 s or those with all options selected in the questionnaire were eliminated, as well as those with regular options.

On the other hand, paper versions of the questionnaires were distributed by trained research staff who confirmed that the respondents met the inclusion criteria and obtained informed consent. The participants were also informed about the precautions for filling out the questionnaires to ensure accuracy and reliability.

### Statistical analysis

Data analysis was conducted using SPSS 27.0 and AMOS 26.0 software. Descriptive statistics such as the means and standard deviations were used for measurement data, while frequencies and percentages were employed for count data. Item analysis was performed using the critical ratio method, correlation coefficient method, and Cronbach’s alpha coefficient method to screen the items for inclusion in the scale. To assess the structural validity of the scale, exploratory factor analysis was used for item screening. Content validity was determined based on expert ratings of the items. Confirmatory factor analysis was utilized to validate the structural integrity of the scale. Additionally, we calculated both convergent and discriminant validity. The reliability of the scale was evaluated using Cronbach’s alpha coefficient, split-half reliability, and retest reliability.

### Ethical considerations

The study received ethical approval from the Ethical Review Committee of Shanxi Bethune Hospital (Shanxi Academy of Medical Sciences) under reference number SBQKL-2022-096. All procedures conducted in studies involving human participants adhered to the ethical standards outlined in the Declaration of Helsinki. Prior to commencing the survey, all nurses were provided with information regarding the study’s objectives and methods. They were informed of their right to withdraw from the study at any point, as well as the handling and confidentiality of the collected data. Nurses who did not agree to participate did not receive the scale, and informed consent was obtained from those who agreed before administering the tool.

## Results

### Initial scale development

#### Results of expert correspondence consultation

In this study, two rounds of expert consultation were conducted with a total of 15 female experts aged between 36 and 53 years (mean age 41.59 ± 6.12 years) and 10 to 25 years of work experience (mean 18.94 ± 7.77 years). Among them, 6 had undergraduate degrees, 7 had master’s degrees, and 2 had doctoral degrees. In terms of professional titles, 3 were intermediate, 9 were associate senior, and 3 were full senior experts. The experts included 5 geriatric clinical nursing experts, 7 geriatric nursing management experts, and 3 geriatric nursing education experts. The effective recovery rate of experts in the two rounds of correspondence was 100%. In the first round, 8 experts proposed revisions (53.33%), while in the second round, 3 experts proposed revisions (20.00%), indicating their high motivation and commitment to the study. The experts’ familiarity with the contents of the inquiry letters in the two rounds (Cs) was 0.729 and 0.727, respectively. The basis of the experts’ judgment (Ca) was 0.893 and 0.900, and the authority coefficients of the experts in the two rounds (Cr) were between 0.052 and 0.315 and 0.000 and 0.236, respectively. The degree of agreement among the experts’ opinions was 0.165 (χ²= 156.038, *P* < 0.001) and 0.195 (χ²= 152.475, *P* < 0.001), respectively, indicating good coordination among the experts.

Based on screening criteria and incorporating feedback from experts and focus group’s discussion, a total of 7 entries were deleted, and 5 entries were modified. The psychological risk dimension lost 2 entries, and the 3rd entry was modified and combined with the physical risk dimension. As a result, the initial scale now comprises 8 dimensions with a total of 45 entries. The main and partial changes were made to the scale: Item 36 “I feel the risk of personal safety due to service distance” was revised to “I feel the risk of personal safety due to long travel distance, inappropriate travel time, and bad travel weather.” Item 43, “Any anxiety, worry, fear in the service process of patient service effect,” was removed because it was deemed similar to risks in hospital care operations that can lead to confusion. Item 44, “Verbal violence that patients or family members may cause to nurses for various reasons,” was merged into the “physiological risk” dimension, as it represents a physiological risk for nurses.

#### Pre-survey results

The nurse proposed modifying A6 from “Patient/family satisfaction with the service effect” to “The service effect is generally consistent with the family/patient’s expectations.”.

### Optimization of scale entries

#### General information of the study population

Through two formal surveys, we gathered data from a total of 8 tertiary hospitals, 3 secondary hospitals, 10 primary hospitals, and 1 Internet + nursing service platform.

that met the inclusion criteria. We collected a total of 492 questionnaires, 464 of which were deemed valid and 29 of which were deemed invalid. For the purpose of screening scale items, 244 samples (initial investigation) were utilized, while 220 samples (the second investigation) were employed for scale validation. It is worth noting that for factor analysis, it is recommended to have a sample size 5 to 10 times the number of items [29]. With 45 entries in the scale, our exploratory factor analysis was conducted with a sample size of 244, meeting the requirements for EFA. The general information of the nurses can be found in Table 1.

**Table 1:** Basic data of the study subjects

Characteristics		First		Second	
		n	%	n	%
sex	Male	6	2.5	13	5.9
	Female	238	97.5	207	94.1
age	≤30	84	34.4	82	37.3
	31-40	122	50.0	117	53.2
	41-50	23	9.4	12	5.5
	≥50	15	6.1	9	4.1
grade of medical institution	Tertiary hospital	240	98.4	137	62.3
	Secondary hospital	2	0.8	32	14.5
	Level hospital	1	0.4	46	20.9
	Network service platform	1	0.4	5	2.3
working life	≤5	81	33.2	60	27.3
	6-10	64	26.2	83	37.7
	11-20	87	35.7	67	30.5
	≥21	12	4.9	10	4.5
positional title	Nurse	50	20.5	50	22.7
	Primary nurse	75	30.7	54	24.5
	Nurse-in-charge	103	42.2	108	49.1
	Deputy chief nurse and above	16	6.6	8	3.7
degree of education	Polytechnic schoosecondary	1	0.6	3	1.4
	Junior college	13	5.4	34	15.5
	Undergraduate course	226	92.6	175	79.5
	Master’s degree or above	4	1.6	8	3.6
marital cstatus	Unmarried	74	30.3	56	25.4
	Married	167	68.5	160	72.7
	Dissociaton	2	0.8	3	1.4
	Bereft of one’s spouse	1	0.4	1	0.5
profession categoryal	Internal medicine	138	56.6	113	51.4
	Surgery	58	23.8	49	22.3
	Emergency call	3	1.2	25	11.4
	Operating room	5	2.0	1	0.5
	Custody room	1	0.4	3	1.4
	Traditional Chinese medicine	36	14.8	9	4.1
	Community branch	3	1.2	20	9.1

#### Results of item analysis

(1) Critical ratio method: The results of the independent sample t test for 45 entry scores between the high and low groups indicated that all entries with *P* < 0.05 were statistically significant and retained. (2) Correlation coefficient method: The correlation between each entry and the total score was analyzed using Pearson correlation. Entries A4, A5, and A7 showed correlations of < 0.4 with the total score, leading to their deletion. (3) Cronbach’s alpha coefficient method: After removing entry A7, the overall Cronbach’s alpha coefficient increased, indicating that this question affected the overall reliability of the scale. Therefore, we decided to delete entry A7. Consequently, the scale comprised 8 dimensions and 42 items.

#### Results of exploratory factor analysis

**Fig. 2:**
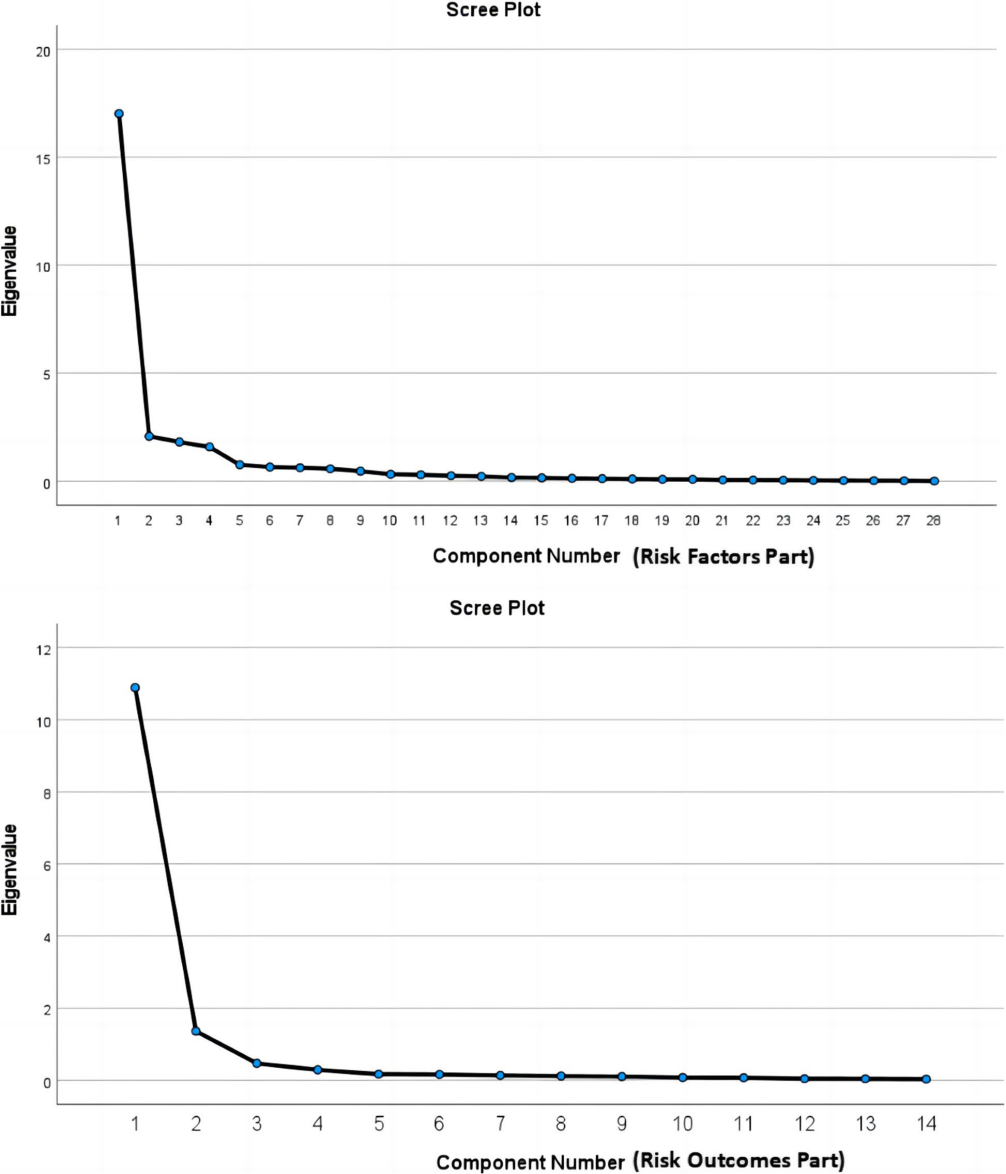
Scree Plot

Since the study evolved into the initial version of the scale in the risk factor part, the items were categorized into 5 distinct groups. Consequently, adhering to the principle of prior decision (a priori criterion), we applied the method of restricting the number of extracted common factors to 5. This decision was made in conjunction with research theory and the study’s objectives. The first exploratory factor analysis showed a KMO value of 0.955 (*p* < 0.001), indicating suitability for factor analysis [22]. Through principal component analysis with rotation using the maximum variance method, we achieved an interpretation rate of 83.137% for cumulative variance. Additionally, when considering the scree plot (refer to Fig. 2) and eigenvalues after the rotation, we determined that the appropriate number of factors for extraction is five. However, entry C16 had factor loadings > 0.5 on both factors, indicating poor discriminant validity, and it was removed. In the second exploratory factor analysis, the KMO value was 0.954 (*p* < 0.001), and the cumulative loading was 84.504%. The rotated matrix of factor loadings confirmed that all entries aligned with the predetermined dimensions, and no entries were deleted. The results of exploratory factor analysis for the risk factors are shown in Table 2.

**Table 2:** Component matrix after rotation

Risk Factors Part					
**Items**	**B**	**C**	**D**	**E**	**A**
B10. The travel tools used on the day of the service are safe.	**0.789**	0.314	0.281	0.170	0.156
B12. The service location is easily identifiable within the community, and transportation around it is convenient.	**0.779**	0.348	0.314	0.122	0.203
B11. The day of service runs smoothly, with convenient transportation.	**0.776**	0.302	0.277	0.199	0.230
B13. The security of the service community is excellent, ensuring environmental safety.	**0.758**	0.350	0.384	0.118	0.181
B14. The family environment of the service recipient is safe, with no obstacles blocking passages or overly slippery floors.	**0.751**	0.387	0.357	0.128	0.202
B9. The distance between the service location and the departure point is appropriate.	**0.724**	0.372	0.213	0.199	0.058
B8. The weather on the day of service is sunny, without snow, fog, windy conditions, or other adverse weather conditions, making it suitable for travel.	**0.718**	0.152	0.264	0.196	0.079
B15. The service recipient’s home and nursing operation environment have sufficient lighting.	**0.700**	0.384	0.381	0.176	0.126
C19. The Internet platform/hospital offers a mobile APP positioning and tracking system, equipped with one-button alarm, delay warning, and other safety devices, to ensure my personal safety.	0.313	**0.832**	0.162	0.287	0.145
C22. The Internet platform/hospital obtains medical liability insurance and personal accident and injury insurance for nurses.	0.312	**0.818**	0.171	0.293	0.160
C18. The Internet platform/hospital can set up work recorders (e.g., video cameras) to ensure service transparency.	0.366	**0.818**	0.178	0.242	0.150
C20. The Internet platform/hospital ensures information and data security, preventing the easy leakage of personal information.	0.355	**0.790**	0.212	0.277	0.233
C21. The Internet platform/hospital offers reasonably priced services.	0.369	**0.762**	0.264	0.197	0.205
C17. Internet platforms/hospitals can ensure the effective utilization of products within their shelf life.	0.482	**0.706**	0.284	0.145	0.175
E28. I am highly proficient in my nursing operation skills.	0.375	0.177	**0.845**	0.210	0.150
E30. I possess the ability to accurately assess clinical problems, address practical nursing issues, deliver health education, and handle emergency treatments.	0.337	0.238	**0.842**	0.243	0.162
E31. When faced with challenging clinical scenarios, I maintain a calm and organized approach, quickly immersing myself in the situation and effectively reflecting on my experiences.	0.345	0.254	**0.836**	0.247	0.150
E29. I excel in communicating with patients and their families.	0.384	0.189	**0.828**	0.243	0.181
E27. My professional knowledge aligns well with the requirements of this service project.	0.374	0.215	**0.803**	0.279	0.172
D26. I have extensive experience in providing “Internet + home care” services, having participated in such projects on three occasions.	0.153	0.381	0.359	**0.759**	0.031
D25. I have undergone systematic and comprehensive training in home care, including relevant “Internet + home care” policies and regulations, complex nursing procedures, emergency nursing responses, interpersonal communication, and professional ethics.	0.266	0.441	0.345	**0.686**	0.114
D24. I am well-versed in the qualifications needed for “Internet + home nursing” service nurses and relevant laws and regulations.	0.235	0.420	0.370	**0.676**	0.178
D23. My medical institution actively encourages “Internet + home care” nurses to offer in-home services.	0.347	0.313	0.342	**0.617**	0.172
A2. The patients and their family members have a high level of education, quality, and refinement.	0.025	0.222	0.230	-0.032	**0.803**
A1. Patients and their family members treat me with politeness and courtesy.	0.041	0.235	0.175	-0.068	**0.782**
A3. I build a trustworthy relationship with the patients and their family members.	0.243	-0.013	-0.028	0.208	**0.719**
A6. The service outcomes are largely in line with the expectations of the family members and patients.	0.288	0.130	0.138	0.251	**0.644**
characteristic root	**6.398**	5.598	5.127	2.883	2.810
Contribution rate of variance(%)	**23.697**	20.733	18.988	10.679	10.406
Cumulative variance contribution rate(%)	**23.697**	44.431	63.418	74.098	84.504
Risk Outcome Part					
Item	Factor Loading				
	Physiological risk		Practice risk	Economic risks	
F37. I have observed instances where patients or family members used false or others’ identity information due to incorrect information from the Internet platform/hospital, resulting in refusal to pay and posing personal safety risks.	**0.879**		0.314	0.253	
F34. In some cases, I noticed the risk of patient or family behavioral violence due to inadequate communication with the patient/family.	**0.867**		0.315	0.297	
F32. I identified the risk of accidental bites or scratches by pets in the patient’s home.	**0.859**		0.273	0.211	
F33. The unsafe factors in the patient’s community and home environment could lead to physical injuries, such as tripping on uneven floors or slipping.	**0.836**		0.295	0.288	
F35. I perceived the risk of verbal violence from patients or their families due to poor communication in certain situations.	**0.806**		0.393	0.289	
F36. I am aware of personal safety risks associated with long travel distances, inappropriate travel times, and adverse weather conditions.	**0.797**		0.395	0.268	
G41. There is a perceived risk of medical disputes arising from emergencies when providing home care without the support of a medical team.	0.313		**0.848**	0.352	
G40. I feel that there might be a risk of medical disputes during home care services due to the patients’ poor physical condition.	0.401		**0.807**	0.377	
G42. I perceive the risk of conducting multisite practices that are not permitted by the law.	0.385		**0.775**	0.415	
G38. I identified the risk of medical disputes resulting from delays or errors in home care due to incomplete or incorrect data collected by Internet platforms/hospitals.	0.458		**0.774**	0.360	
G39. I perceive the risk of medical disputes in home care due to the unreasonable use of resources (e.g., insufficient or expired supplies) provided by the Internet platform/hospital.	0.446		**0.738**	0.410	
H43. I am concerned about the risk of economic damage due to imperfect payment operations or supervision by Internet platforms/hospitals, such as theft of account and password.	0.343		0.468	**0.769**	
H44. I observed the potential risk of financial damage resulting from punitive measures (e.g., bonus deductions) when the medical institution does not endorse nursing staff engaging in home-based care work outside of the facility.	0.361		0.449	**0.759**	
H45. I have apperceived the risk of economic harm arising from the disparity between the service time or economic cost and the income generated from the service.	0.334		0.469	**0.735**	
characteristic root	5.421		38.721	38.721	
Contribution rate of variance(%)	4.425		31.604	70.325	
Cumulative variance contribution rate(%)	2.880		20.571	90.896	

The risky ending section was limited to 3 public factors based on the predefined dimensions according to the law of prior decision. The exploratory factor analysis showed a KMO value of 0.945 (p < 0.001), indicating suitability for factor analysis. The cumulative loading was 90.896%, and the factor loading rotation matrix confirmed that all entries were in the predetermined dimensions with no deletions. As a result, the final test version of the scale comprised 8 dimensions and 41 entries. The retained dimensions and entries were relatively evenly distributed, which was in line with the original theoretical hypothesis. The scree plot (refer to Fig. 2) and the results of exploratory factor analysis for the risk outcome sections and are also presented in Table 2.

### Test of scale measurement properties

#### General information of the study subjects

A total of 232 questionnaires were collected through the online and paper versions in the second suivey. After excluding 12 invalid questionnaires, the final sample size was 220. Forty-two entries were employed for the confirmatory factor analysis, and this analysis was conducted with a sample size of 210 at least, meeting the requirements for CFA. The general information of the study participants is presented in Table 1.

#### Reliability assessment

Cronbach’s α coefficient was found to be 0.970, indicating high internal consistency, and the half reliability was 0.876. The individual Cronbach’s α coefficient and half reliability for each dimension are provided in Table 3. The retest reliability was 0.980, demonstrating good stability over time, and the correlation ranged from 0.510 to 0.996 (*P* < 0.01).

**Table 3:** Scale of aggregate validity

Factor	Item	Standardized Factor Loading	S.E.	P	CR	AVE	Cronbach’s Alpha	Half reliability
A. Patient/family side risk assessment	_A1_	0.803			0.809	0.515	0.803	0.801
	_A2_	0.675	0.095	***				
	_A3_	0.706	0.102	***				
	_A4_	0.68	0.098	***				
B.External environmental risk assessment	_B5_	0.647			0.963	0.765	0.961	0.945
	_B6_	0.899	0.126	***				
	_B7_	0.865	0.125	***				
	_B8_	0.94	0.125	***				
	_B9_	0.939	0.124	***				
	_B10_	0.954	0.124	***				
	_B11_	0.868	0.125	***				
	_B12_	0.845	0.126	***				
C.Platform/hospital supply risk assessment	_C13_	0.811			0.924	0.668	0.922	0.893
	_C14_	0.811	0.057	***				
	_C15_	0.797	0.081	***				
	_C16_	0.824	0.065	***				
	_C17_	0.856	0.062	***				
	_C18_	0.805	0.077	***				
D.Nurse service related experience	_D19_	0.775			0.894	0.680	0.889	0.862
	_D20_	0.876	0.087	***				
	_D21_	0.888	0.092	***				
	_D22_	0.751	0.103	***				
_E.Nurses of core clinical competence_	_E23_	0.915			0.973	0.880	0.973	0.925
	_E24_	0.947	0.039	***				
	_E25_	0.934	0.041	***				
	_E26_	0.944	0.039	***				
	_E27_	0.949	0.04	***				
F.Physiological risk	_F28_	0.828			0.949	0.755	0.947	0.929
	_F29_	0.902	0.057	***				
	_F30_	0.935	0.056	***				
	_F31_	0.834	0.058	***				
	_F32_	0.814	0.066	***				
	_F33_	0.895	0.061	***				
G.Practice risk	_G34_	0.924			0.949	0.787	0.948	0.891
	_G35_	0.916	0.044	***				
	_G36_	0.921	0.047	***				
	_G37_	0.856	0.055	***				
	_G38_	0.814	0.059	***				
H.economic risk	_H39_	0.911			0.925	0.804	0.922	0.825
	_H40_	0.921	0.048	***				
	_H41_	0.857	0.056	***				

*AVE *Average variance extracted, *CR *Construct reliability, *S.E. *Standard error

#### Validity assessment

##### Content validity

Six nursing experts and two geriatricians were invited to assess the scale. The results indicated that the I-CVI ranged from 0.875 to 1.000, and the S-CVI/Ave was 0.941, demonstrating good content validity for the scale.

##### Structural validity validation

**Fig. 3:**
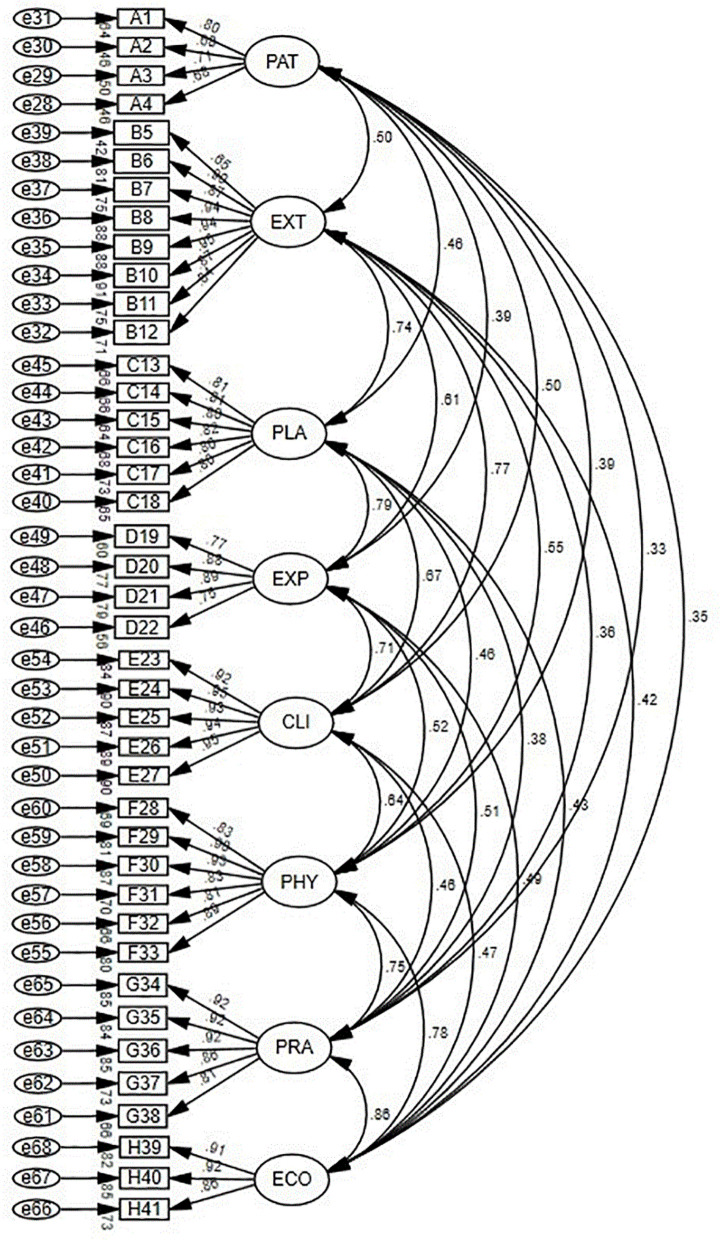
Model fitting diagram. (PAT: Patient/family side risk assessment; EXT: External environmental risk assessment; PLA: Platform/hospital supply risk assessment; EXP: Nurse service related experience; CLI: Nurses of core clinical competence; PHY: Physiological risk; PRA: Practice risk; ECO: Economic risk.)

In this study, the model showed good fit with χ2/df = 2.086, RMR = 0.045, RMSEA = 0.070, CFI = 0.918, TLI = 0.911, and IFI = 0.919, all falling within an acceptable range. Furthermore, the correlation coefficients between the scale entries and their respective domains were found to be between 0.65 and 0.93 (p < 0.01), providing further evidence for the reasonable structural validity of the scale. The model fit diagram is displayed in Fig. 3.

##### Convergent validity

To assess the convergent validity of the scale, both CR and AVE were examined. The CR values for each dimension ranged from 0.809 to 0.973, and the AVE values ranged from 0.515 to 0.880. These results met the criteria of CR > 0.7 and AVE > 0.5, indicating that the scale demonstrated good convergent validity. Detailed values can be found in Table 3.

##### Discriminant validity

The square root of AVE for each dimension is higher than the correlation between that dimension and other dimensions, indicating good discriminant validity. Table 4 shows the detailed results.

**Table 4:** Scale for discriminant validity

Factor	A	B	C	D	E	F	G	H
A	0.717							
B	0.504***	0.874						
C	0.461**	0.743**	0.818					
D	0.386**	0.612**	0.789**	0.825				
E	0.502**	0.770**	0.667**	0.709**	0.938			
F	0.394**	0.547**	0.464**	0.523**	0.644**	0.869		
G	0.330**	0.361**	0.379**	0.508**	0.459**	0.747**	0.887	
H	0.347**	0.419**	0.432**	0.488**	0.471**	0.783**	0.863**	0.896

The diagonal number is the square root of AVE values in dimension. **p<0.01, ***p<0.001. A: Patient/family side risk assessment; B: External environmental risk assessment; C: Platform/hospital supply risk assessment; D: Nurse service related experience; E: Nurses of core clinical competence; F: Physiological risk; G: Practice risk; H: Economic risk

## Discussion

### Innovative and meaningful scale development

Currently, in Chinese “Internet + nursing service,” risk assessment tools for nurses mainly consist of indicator systems and questionnaires. However, there is no locally developed scale specifically designed to assess the risks faced by nurses during the service process. For this reason, Yanhong Du [30] devised a risk perception questionnaire targeting online nurses. This questionnaire assesses the frequency of perceived risk among nurses, encompassing different levels of risk perception (ranging from “never” to “almost always”). The study also investigates how risk perception relates to psychological influences, resilience, and emotions. To identify relevant risk factors in the service process, the study employs the 5M1E field management theory, which captures multidimensional risk factors associated with the service process. Unlike prior research that focused on unidimensional aspects such as demographic information and psychological traits, this study emphasizes objective risks faced by nurses concerning people and the environment during service delivery. The assessment of the degree of risk perception, ranging from “almost never” to “extremely high,” offers an innovative approach that enriches research in this area from a different perspective.

This research employs the 5M1E method and risk perception as the theoretical framework to explore the risks faced by nurses in “Internet + Nursing Service.” The risk factor section includes dimensions such as “patient/family risk assessment” and “external environment risk assessment,” which align with the risk concerns identified in qualitative interviews by Liu Chao et al. [31]. These dimensions have also been highlighted by scholars both domestically and internationally as important areas of focus [32–34]. In the risk endings section, we prioritize nurses’ perspectives by considering their risk perceptions rather than relying on managers’ and researchers’ summaries from a third-party standpoint. By doing so, we determine whether specific risks have occurred and assess the level of perceived risks from nurses’ viewpoints.

This approach allows us to address nurses’ safety needs from multiple dimensions and provide a basis for identifying relevant risks. The primary aim of this study is to evaluate the risk factors and risk outcomes experienced by nurses during “Internet + Nursing Service” for elderly individuals. This assessment aids in the early identification of risks and serves as a foundation for developing risk warning models and safety systems. Furthermore, it provides clinical managers with insights to formulate effective risk countermeasures. Ultimately, this research contributes to reducing nurses’ risk concerns and promoting their active participation in providing services. The practical significance of this study is substantial.

### The reliability of the assessment scale

As per the literature, the scale’s internal consistency should be greater than 0.7, the half reliability should exceed 0.7, and the correlation coefficient of retest reliability should be higher than 0.7 [24]. In this study, Cronbach’s α for the “Internet + Nursing Services for the Elderly” Nurse Risk Factor and Risk Outcome Assessment Scale was found to be 0.970, with each dimension ranging from 0.803 to 0.973. These values indicate strong internal consistency for the scale. Additionally, the split-half reliability for the total scale was 0.876, with each dimension ranging from 0.801 to 0.945, further confirming good reliability. The retest reliability for the total scale was 0.980 (*p* < 0.01), and for each dimension, it ranged from 0.510 to 0.996, all of which were statistically significant. In conclusion, the scale demonstrates good reliability.

### The validity of the assessment scale

The scale’s content validity generally requires I-CVI ≥ 0.78 and S-CVI/Ave ≥ 0.9.

In this study, the I-CVI ranged from 0.875 to 1.000, and the S-CVI/Ave was 0.942 based on expert ratings, indicating good content validity. The exploratory factor analysis aligned with the predefined dimensions. Confirmatory factor analysis showed that the entries in the scale had significant correlations (ranging from 0.65 to 0.93, *p* < 0.01) with their respective domains. The fit indices χ2/df = 2.086, TLI = 0.911, CFI = 0.918, RMR = 0.045, and RMSEA = 0.070 indicated a good and acceptable model fit. Moreover, the standardized factor loadings for all items were > 0.4, demonstrating favorable structural validity. The scale exhibited good convergent validity (CR > AVE for each dimension) and good discrimination (the square root of AVE for each dimension > the correlation with other dimensions). Hence, the scale demonstrated good validity.

## Conclusion

To summarize, the “Internet + nursing services” nurse risk factor and risk outcome assessment scale for the elderly developed in this study showed good reliability and validity. It comprises two parts: risk factors and risk outcomes for nurses, with a total of 8 dimensions and 41 items (the final version of the scale can be found in the supplementary materials). The scale is suitable for assessing nurses’ involvement in Internet + nursing services within the cultural context of China. However, it is important to note that this study utilized a convenience sampling method, with survey respondents mostly being nurses from internal medicine departments in tertiary hospitals who participated in “Internet + nursing services” for elderly individuals. Therefore, further research is needed to examine the applicability of the scale to different hospital levels and departments that may have different service targets, not limited to the elderly population.

## Data Availability

The datasets generated and analyzed during the current study are not publicly available due to subsequent analysis of the project to construct the early warning model but are available from the corresponding author upon reasonable request.
